# Mouse Exploratory Behaviour in the Open Field with and without NAT-1 EEG Device: Effects of MK801 and Scopolamine

**DOI:** 10.3390/biom14081008

**Published:** 2024-08-15

**Authors:** Charmaine J. M. Lim, Jack Bray, Sanna K. Janhunen, Bettina Platt, Gernot Riedel

**Affiliations:** 1 Institute of Medical Sciences, University of Aberdeen, Aberdeen AB25 2ZD, UK; limcharmainejm@hotmail.com (C.J.M.L.); r05jb17@abdn.ac.uk (J.B.); b.platt@abdn.ac.uk (B.P.); 2 Organon R&D Finland, Itäinen Pitkäkatu 4, 20520 Turku, Finland; sannajanhunen@hotmail.com

**Keywords:** reproducibility, open field test, electroencephalography, wireless, MK801, scopolamine

## Abstract

One aspect of reproducibility in preclinical research that is frequently overlooked is the physical condition in which physiological, pharmacological, or behavioural recordings are conducted. In this study, the physical conditions of mice were altered through the attachments of wireless electrophysiological recording devices (Neural Activity Tracker-1, NAT-1). NAT-1 devices are miniaturised multichannel devices with onboard memory for direct high-resolution recording of brain activity for >48 h. Such devices may limit the mobility of animals and affect their behavioural performance due to the added weight (total weight of approximately 3.4 g). The mice were additionally treated with saline (control), N-methyl-D-aspartate (NMDA) receptor antagonist MK801 (0.85 mg/kg), or the muscarinic acetylcholine receptor blocker scopolamine (0.65 mg/kg) to allow exploration of the effect of NAT-1 attachments in pharmacologically treated mice. We found only minimal differences in behavioural outcomes with NAT-1 attachments in standard parameters of locomotor activity widely reported for the open field test between the drug treatments. Hypoactivity was globally observed as a consistent outcome in the MK801-treated mice and hyperactivity in scopolamine groups regardless of NAT-1 attachments. These data collectively confirm the reproducibility for combined behavioural, pharmacological, and physiological endpoints even in the presence of lightweight wireless data loggers. The NAT-1 therefore constitutes a pertinent tool for investigating brain activity in, e.g., drug discovery and models of neuropsychiatric and/or neurodegenerative diseases with minimal effects on pharmacological and behavioural outcomes.

## 1. Introduction

With advancements made in preclinical research, laboratories often align the mechanistic and functional aspects of animal behavioural outcomes with those of dynamic neuronal events. This is often performed with tethered or, more recently, wireless miniature recording systems [1,2,3,4,5,6,7,8] to allow recording of electroencephalography (EEG) in freely moving animals with minimal discomfort. Other devices and their properties have been reviewed recently [9]; there are a limited number of data logging devices with onboard memory (Deuteron mouse-log16 and TSE Neurologger) that are similar in weight to the NAT-1 (~3 g) but differ for applications and sampling rates. Most devices are battery-powered high-weight transmitters (Neuralynx, Pinnacle; up to 23 g) with continuous data download but limited battery life. Most of these devices are not applicable to mice and are used for the recording of single units or single cells. In our laboratory, the use of an EEG wireless device includes the bilateral implantation of gold electrodes into the skull and the attachment of a lightweight neural activity tracker (NAT-1; Cybula, York, UK) device to this head stage prior to behavioural testing. These devices have been employed for sleep-staging and global activity analysis during home cage recordings [7,8]. However, the surgical procedure and additional weight a mouse must tolerate amounts to about 3 g (multifunctional NAT-1 device including batteries), in addition to a small head stage modelled from dental cement and screw recording electrodes. The impact on the animal’s head could therefore conceivably limit locomotor skills and the mobility of the animal and affect its natural explorative behaviour, and consequently the outcomes of behavioural findings, thereby altering the apparent effects of pharmacological agents.

Compounds commonly used in preclinical in vivo research such as N-methyl-D-aspartate receptor (NMDAR) antagonist (dizocilpine; [+]-MK801) and muscarinic antagonist, scopolamine, were inoculated in mice in this study. Both compounds induce schizophrenia- [10,11,12,13] or Alzheimer-related [14,15] cognitive decline respectively and are known to induce well measurable and robust behavioural outcomes in mice including dose-dependent hyperactivity/hypoactivity [13,14,15,16,17,18,19]. Inclusion of prespecified doses of MK801 and scopolamine (unpublished preliminary data) in this study will allow opposing nonsystematic movements in mice to accurately represent the effects of wireless recording device attachments in animals.

This exploratory study aims to: (1) evaluate potential differences in behavioural outcomes in the open field test in mice with and without surgically implanted head stages/EEG recording devices (NAT-1); and (2) explore the behavioural effects of such attachments in pharmacologically treated mice. Here, the open field test was the selected behavioural paradigm as it produces sufficiently reliable and repeatable measures under standardised conditions. All exploratory behaviour obtainable from video-observed tests are presented to avoid bias; EEG recordings obtained in this study were not deemed relevant to changes in behavioural outcomes in mice and are therefore omitted. Generally, analyses of activity in the open field test confirm that despite very similar performance with or without EEG devices in vehicle-treated control mice, subtle differences appeared in the drug groups that may inform of some behavioural alterations that affect outcomes other than activity.

## 2. Methods

### 2.1. Experimental Design

The study design, analysis, and reporting methods in this study were conducted in line with recommendations stated in the ARRIVE 2.0 guidelines [20] and are detailed in the relevant segments below. The study design comprised of two independent experiments conducted on separate occasions. The testing days of the week, number of animals tested per day, and testing seasons were, however, similar between the studies. To ensure that any differences between Experiments 1 (no NAT-1) and 2 (with NAT-1) were the result of the attachment of wireless data logging devices but not that of variations in procedural protocols or testing environment, special care was taken to ensure that the laboratory conditions, husbandry, avoidance of bias, and study designs were maintained between both experiments (detailed in Table 1). Variables which differed between Experiments 1 and 2 are marked with asterisks in Table 1 and mainly pertained to the surgical procedures and NAT-1-related handling requirements performed in Experiment 2.

### 2.2. Animals

Twenty-nine C57BL/6J (10–11-week-old male mice) from Charles River Laboratories (Kent, England) were obtained for Experiment 1 and 37 for Experiment 2. The mice were housed individually in standard Macrolon shoebox cages (Techniplast 1145) measuring at 369 x 165 x 132 mm, under a 12-h light/12-h dark cycle (lights on at 7 am). Temperature and humidity were set at 20 ± 5 °C and 48 ± 5% in both the housing and experimental rooms. The animals had free access to standard rodent food chow (SDS 801190 RM3, Special Diet Services (SDS)/Augy/France) and water ad libitum. All the animals were acclimatized to the facility environment and extensively handled for 2 weeks prior to the start of the surgeries and/or open field testing. The mice were tail-handled to avoid detachment of the head stages and management of severe stereotypic responses during transfer. All housing and handling of the animals were in accordance with the international standards on animal welfare (EU/63/2010) and Home Office (UK) regulations. At the end of the study, all the animals were euthanised and the brain tissue harvested.

### 2.3. Stereotactic Surgery and Home-Cage Recordings (Experiment 2 Only)

The animals had free access to water supplemented with carprofen Rimadyl^®^ (5 mg/kg; Zoetis, UK) and were acclimatised to soft diet (Transbreed (E), SDS 801222, England; and Solid Drink^®^ SDST-75, 3at-bio, Tiel, The Netherlands) 48 h prior to and after surgery. Stereotaxic surgeries were conducted as described previously [3,7,8,21]. Briefly, anaesthesia was induced with 3% isoflurane (IsoFlo^®^, Zoetis) in medical-grade oxygen and maintained on 1.5% isoflurane during surgery. The mice were held in a stereotaxic frame (Stoelting, Wood Dale, IL, USA). Epidural gold-plated screw electrodes were placed at the following stereotaxic locations [22] on exposed skulls from the prefrontal cortex (2 mm anterior to bregma/close to midline), left and right hippocampus (2 mm posterior to bregma, 1.5 mm lateral to midline). Reference and ground electrodes were placed in a neutral location above the occipital plate. The electrodes were soldered and assembled into a 6-pin adaptor and fixed on to the skull by a mixture of Durelon^®^ dental cement and tissue adhesive. The pins measured 0.40 mm (square) with a spacing pitch of 1.27 mm. Once the dental cement dried, the animal was removed from the stereotaxic instrument and injected with 1.0 mL saline (intraperitoneal) and 0.2 mL of 0.1 mg/kg buprenorphine (subcutaneous; Vetergesic^®^, Cera Animal Health Ltd., High Wycombe, UK). The animals were placed on heating pads immediately after the procedure, and further analgesic treatment continued for 2–3 days as appropriate. Following surgery, the animals were weighed daily to monitor their recovery and were provided with a soft diet. A minimum of 7 days was allowed for recovery before the start of testing. Ten animals were not entered into this study due to dislodged head caps and were euthanised. From these, 6 head caps were lost during restraining for drug administration or NAT-1 attachment/removal, while an additional 4 lost their head stages in their home cages presumably while getting stuck in the wire tops. We have since then modified this procedure and hold animals in individually ventilated cages now. After the open field testing, all the operated mice went on to be tested for home cage activity and were finally euthanised to confirm electrode placement (not relevant here).

### 2.4. Drugs and Drug Formulation

The drug and dose selection were in line with the EQIPD project (see funding statement), in which between-laboratory robustness of the data was proposed and both drugs were utilized for drug comparison [23], but also for EEG recordings between laboratories [24].

(+)-MK801 maleate (CAS: 77086-22-7) and scopolamine hydrobromide (CAS: 114-49-8) were obtained from Tocris Bioscience (Bristol, UK) and diluted to 0.65 mg/kg MK801 and 0.85 mg/kg scopolamine (salt-corrected for both drugs) in 0.9% saline 24 h prior to intraperitoneal injections. Saline was administered as vehicle control. Reagents were administered (10 mL/kg) as a single dose, with a sterile injection needle, 30 min prior to the behavourial testing. The assignment of all the mice to different drugs was randomised (Experiment 1: n = 10 for saline, n = 9 for MK801, and n = 10 for scopolamine; Experiment 2: n = 9 per drug group) using a Williams Square design [25].

### 2.5. Apparatus and Testing Protocols

Upon behavioural testing in the open field arena, the mice were aged 12–13 weeks old and weighed approximately 26.8 ± 2.0 g. Intraperitoneal administration of drugs and testing sequence were conducted in a blinded manner. Common to both experiments, the mice were habituated to the test room for 30 min, following drug inoculations. In Experiment 2, the mice were drug-treated immediately before the attachment of the NAT-1 devices. NAT-1 measures 18 × 22.2 × 10.3 mm and weighs approximately 2.2 g including a zinc battery (as). The total weight of the NAT-1 and head stage (ranging between 1.1 and 1.5 g) equals approximately 3.4 g (Figure S1). The attachment of the wireless NAT-1 device to the head stage involved temporarily restraining the mice between two pieces of faux fur fabric. This restraint is necessary to minimise movement and to ensure the secure placement of the device with minimal discomfort imposed on the mouse. The restraint was performed by the same trained experimenter throughout the study. A second trained experimenter carefully removed the pin protection and slid the NAT-1 onto the head stage. The entirety of the process generally took about 20 s.

To quantify the behavioural outcomes of using the NAT-1, we tested the activity of all the mice in the open field test [26,27,28]. The open field tests were conducted in a dedicated sound-attenuated room and in a square box (made up of white reflective Perspex) of 50 × 50 × 40 cm (length × width × height). Two white LED lights facing upwards from the arena and the main fluorescent room lights were switched on, giving rise to an average illumination of 320 lux. Temperature and humidity were maintained at 20 ± 5 °C and 48 ± 5%, respectively. An overhead camera (Imaging Source, DMK22AUCO3, Bremen, Germany), 125 cm from the floor of the arena, was connected to an automated recording system (ANY-Maze v. 5.1, Stoelting) for online tracking of the animal’s centre of mass. Each mouse was placed into the centre of the open field and allowed to freely explore for 30 min, and the apparatus was thoroughly cleaned with odourless wet wipes between each mouse. The automatic tracking option was defaulted in ANY-Maze to allow the software to adjust tracking parameters by contrasting the apparatus background, illumination, and animal coat colour. Sampling rates were maintained at the programme’s recommended 15 frames per second (fps) for small animals. All the analyses were conducted offline with the same software (ANY-Maze v. 6.1). Parameters which were widely recorded by different laboratories (see [29] for review) were explored. These included the following:

- *total distance* travelled in the arena;
- *line-crossings* in a 16-grid overlay;
- *meandering* (a measurement of an animal’s *absolute turn angle* over the *total distance travelled*, which refers to the winding movement of animal);
- *frequency of rotations* (total of 360 degree turns to the left and 360 degree turns to the right);
- degree of thigmotaxis (a ratio of distance travelled in the periphery zone against the total distance travelled);
- *average distance from the centre point* (to provide meaningful indications of the general location of the mice without utilizing zonal outlines).

Thigmotaxis zone was defined as the zone 5 cm from the arena walls.

### 2.6. Exploratory Data Analyses

Representative track plots of the animals in each drug group of Experiments 1 and 2 were generated by extracting pixel values of the animal’s centre point from ANY-Maze and converting them to millimetres using a MATLAB script. The track plots were further divided into two-time intervals (0–15 min and 15–30 min) for clarity and plotted using MATLAB (R2019a, Mathworks, Natick, MA, USA).

Additional exploratory analyses of all the outcomes obtainable from ANY-Maze were conducted to minimise and avoid hypothesis-driven selection of parameters in the open field test [30] by means of heat mapping [31,32]. A total of 68 analysis parameters were extracted by ANY-Maze and were listed for comparison. Heat maps were created to show all data points for the comparison of effects of NAT-1 on each drug condition. The heat maps were generated with *p*-values obtained from independent two-tailed *t*-tests with Satterthwaite correction for heteroscedasticity and bootstrapped 1000 times with replacement (seed set at 123456). The intensities of colours on the heat map were scaled according to the *p*-value threshold set at 95% confidence level. Parameters in the heat maps were clustered for clarity. *Apparatus measures* (#1–20) concerning *total distance* and other additional information regarding activity for the entirety of the test were categorized at the top of the map. *16-grid crossings* (#21), *centre-point* measures (#22–29), and *thigmotaxic zone* measures (#30–68) which are generally used as *standard parameters* were clustered next (Table 1). Heat maps represent global readouts over 30 min, so time-dependent differences and scoring by *segment of test* were not considered. *Immobility* and *freezing* parameters were defaulted in ANY-Maze as 2000 milliseconds (sensitivity level set at 65%) and 1000 milliseconds (threshold levels on at 30 and off at 40) as the minimum immobility and freeze durations, respectively, and no changes were made to either of the criteria. Parameters from head and tail tracking were not recorded.

### 2.7. Statistical Analysis

Parameters of interest obtained from the tracking software and calculated parameters of interest (e.g., total rotations, meandering, and thigmotaxis) were first tested for normality using the Lilliefors Kolmogorov-Smirnov test. The observed unpaired mean differences were reported and derived from the expected sampling error to illustrate the magnitude of differences using estimation analysis [33]. *t*-tests or analysis of variance (ANOVA; for total responses parameters) with post hoc Bonferroni’s corrections were performed for all the conventional p-value analyses and bootstrapped 1000 times with replacement (seed set at 123456) to accommodate non-normally distributed outcomes [34]. Group differences were also considered negligible if the effect sizes (ηp^2^) were less than 0.1. Only data for distance moved for the entirety of the test were further collapsed into 5 min bins; statistical differences were evaluated using two-way ANOVA for repeated measures, with experimental groups as between-subject factors and time as within-subject factors. Statistical significance was set at 95% confidence level for the conventional statistics. Outliers (with residuals more than two standard deviations away from the mean) were re-investigated for extreme experimental issues or recording errors. No convincing reasons to remove any data on this basis were found. All conventional analysis, analysis of estimates, and visualisation of outcomes were performed in R (v.4.3.0; [35]).

## 3. Results

**Figure 1:**
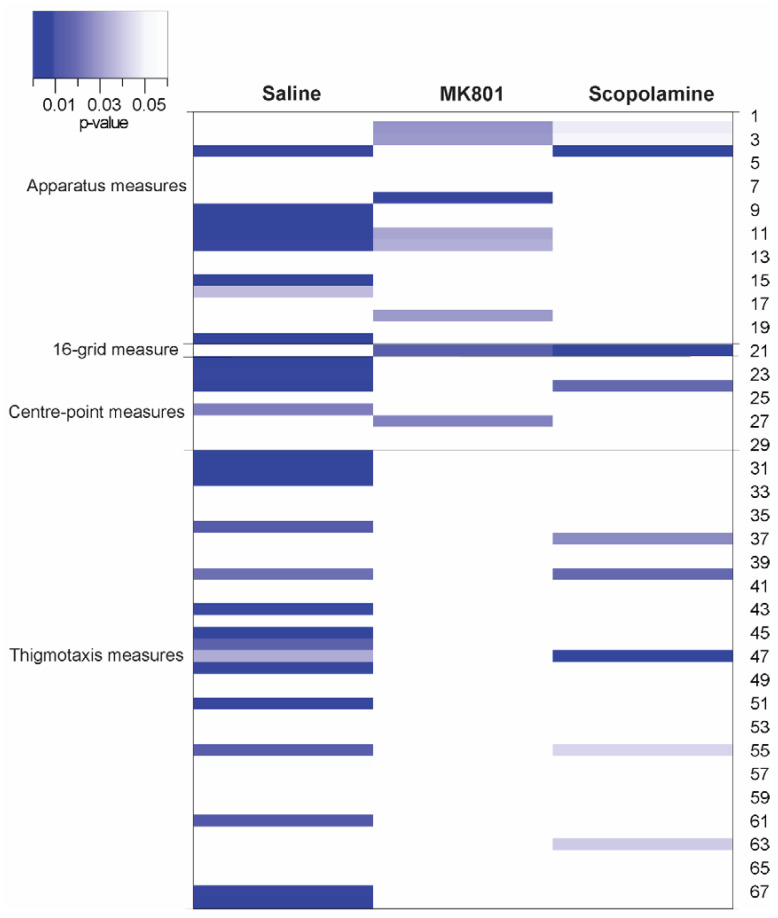
Heat map comparing experiment 1 (no head stage/NAT-1) and 2 (with head stage + NAT-1) for each drug treatment. Parameters are clustered into *apparatus measures* (#1–20; commonly used in the analysis of standard parameters); *16-grid measure* (#21); *centre-point measures* (#22–29); and *thigmotaxis measures* (#30–68). The intensity of colours on the heat map was scaled accordingly with the head stage/treatment cohorts, and the parameter presenting the greatest differences of *p*-values between head stage/treatment cohorts were isolated and analysed. Data presented in blue in the heat map represent a *p*-value with the threshold of 0.05 in the between-experiment comparisons. The heat map was visualized in R (v4.3.0).

The experimental and housing conditions for both independent experiments are detailed in Table 1; the surgical procedures and NAT-1 attachments were the only inconsistent variables between both experiments. Comparisons for each drug treatment were initially visualised in a heat map (Figure 1); significant differences where *p* < 0.05 thresholded are represented in blue. Head stage/NAT-1 attachments led to significant differences in 32 parameters for the saline-treated groups, 12 parameters for the MK801-treated groups, and 14 parameters for the scopolamine-treated groups. No parameter showed a statistical significance in all the comparisons between experiments for all the treatments (Figure 1 and Table S1).

**Figure 2:**
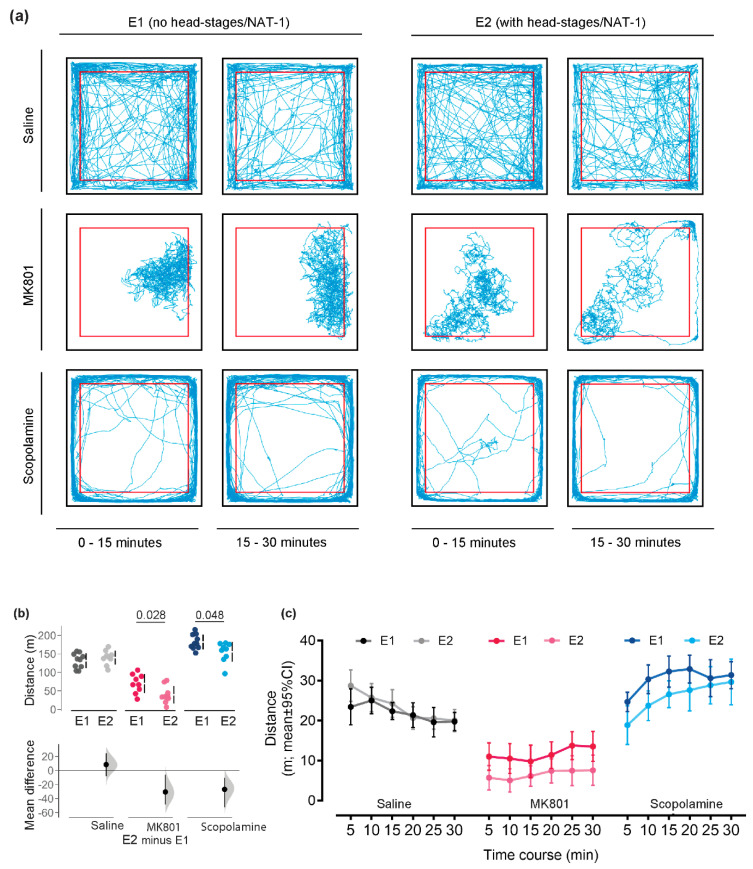
Behavioural responses for locomotor activity between Experiments 1 (E1) and 2 (E2). (**a**) Representative exploration paths of animals (with and without head stage + NAT-1) administered with vehicle, MK801 or scopolamine recorded in the open field test and visualised by track plots divided into time bins, 0–15 min and 15–30 min. The *periphery* (thigmotaxis zone) of the open field arena is demarcated by a red border, 5 cm away from the walls of the arena. Representative paths were illustrated using an in-house MATLAB (R2018a, Mathworks, USA) script. (**b**) Analysis of locomotor activity by *cumulative distance travelled* and (**c**) *total distance travelled* over 30 min of testing in the open field was visualised in 5 min time bins. Individuals are represented by single data points with their respective means (gap) ± standard deviation (vertical lines) in the top axis. The shaded curve and the error in the bottom axis demonstrate the distribution of sampling error and its respective 95% confidence interval for the difference between the means. Performance of animals in E1 is represented by the 0 line in the bottom axis. Conventional statistics (independent sample *t*-tests) revealing a significance set at 95% (*p* < 0.05) are annotated as text on (**b**). All analyses were bootstrapped at 1000 resamples, with seed set at 123456 and visualized in R (v.4.3.0). E1, with no head-stage/NAT-1; and E2with head-stage and NAT-1.

The explorative strategies were visualised by track plots of representative animals in Experiments 1 and 2 (Figure 2a). Locomotor activity and spatial distribution in the open field were maintained between animals with or without NAT-1 of the same drug treatment in both the first and second 15 min interval of the experiment. For both experiments, the scopolamine-treated animals remained in the *periphery* whilst the MK801-treated animals remained in a section of the arena, generally localised away from the arena walls. The saline-treated animals traversed across the centre with a greater frequency than the two drug groups and hence did not show a clear preference for an exploration zone.

The head-stage/NAT-1 attachments decreased *total distance travelled* by 30.6 m [95% CI −48.6, −6.04 m] and 26.9 m [95% CI −52.3, −10.4 m] in the MK801-and scopolamine- treated animals, respectively. The means of *total distance travelled* were outside of the interval estimate but were minimally increased by 8.37 m [95% CI −8.09, 24.6 m] in the saline-treated group (Figure 2b). The *Total distance travelled* analysed in 5 min time intervals are visualised in Figure 2c. No main effect of head stages was observed for the saline treatments, but significant reductions were yielded for the MK801- (F(1,16) = 6.98, *p* = 0.018, ηp^2^ = 0.304) and scopolamine- (F(1,17) = 6.26, *p* = 0.023, ηp^2^ = 0.269) treated cohorts.

**Figure 3:**
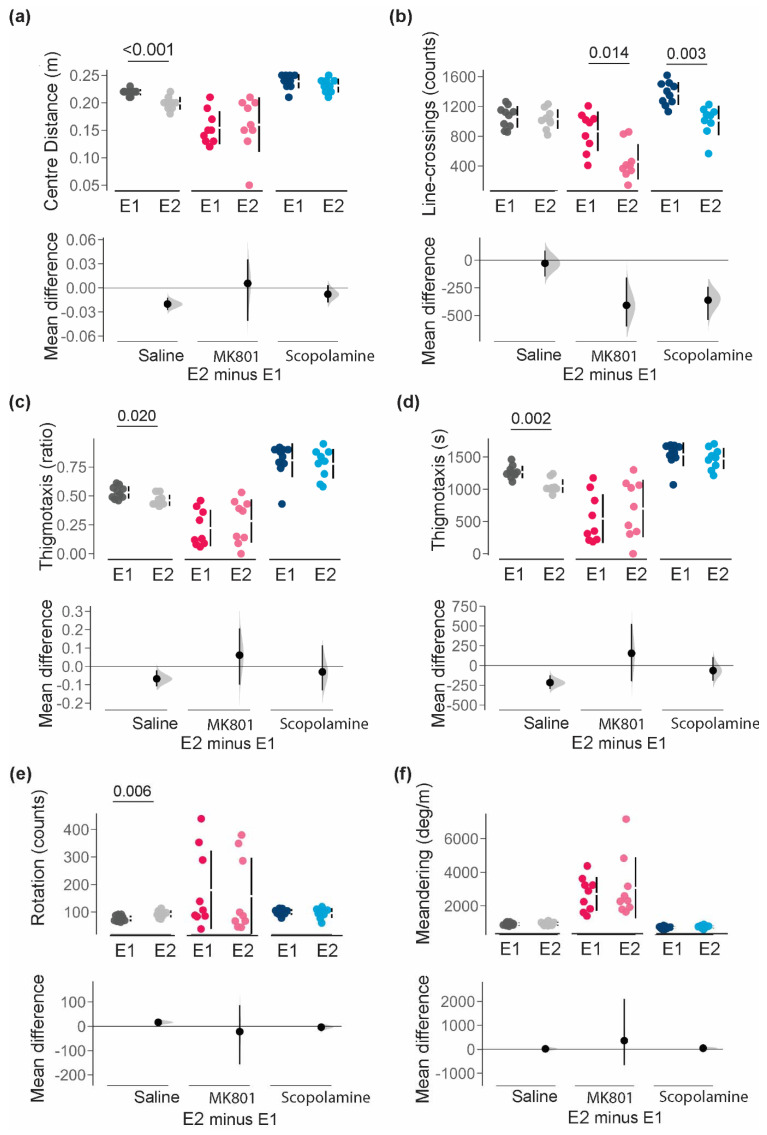
Comparison of behavioural responses for parameters of distance between Experiments 1 (E1) and 2 (E2). (**a**) *average distance from the centre-point*; (**b**) *line-crossings*; (**c**) *thigmotaxic ratio*; (**d**) *time spent in the thigmotaxic zone*; (**e**) *total number of rotations*; and (**f**) *meandering*. Individuals are represented by single data points with their respective means (gap) ± standard deviation (vertical lines) in the top axis. The shaded curve and the error in the bottom axis demonstrate the distribution of sampling error and its respective 95% confidence interval for the difference between the means. Performance of animals in E1 is represented by the 0 line in the bottom axis. Conventional statistics (independent sample *t*-tests) revealing significance set at 95% (*p* < 0.05) are annotated as text on the graphs. All analyses were bootstrapped at 1000 resamples, with seed set at 123456, and visualized in R (v.4.3.0). E1 with no head-stage/NAT-1; and E2 with head-stage and NAT-1.

Comparisons of animals with and without NAT-1 are statistically summarised in Table 2 and visualised in Figure 3. No notable differences due to the NAT-1 attachments were discerned in the *average distance from the centre-point* (m [95% CI]: saline, −0.020 [−0.026, −0.012]; MK801, 0.006 [−0.041, 0.036]; and scopolamine, −0.080 [−0.018, 0.030]); Figure 3a). *Line-crossings* differed between experimental groups for all treatments (Figure 3b). NAT-1 attachments induced the largest reductions in line-crossing counts in the MK801- (−409 counts [95% CI, −600, −158 counts]) and the scopolamine- (−362 counts [95% CI, −541, −242 counts]) treated animals, but minimally affected the saline-treated mice (−28.6 counts [95% CI, −148, 86.7 counts]).

In parameters of localization, head-stage/NAT-1 attachment in animals was estimated to decrease *thigmotaxis ratio* and *thigmotaxis time* in saline-treated animals (−0.068 [95% CI, −0.11, −0.021] and −216 s [95% CI, −295, −123 s] respectively), but not under exposure to MK801 or scopolamine (Figure 3c,d). Comparisons between Experiments 1 and 2 also confirmed minimal differences in *rotations* (counts [95% CI]: saline, 16.3 [5.6, 26]; MK801, −21.9 [−156.0, 87.5]; and scopolamine, −3.7 [−18.9, 7.16]) or *meandering* (degrees/m [95% CI]: saline, 19.5 [−63.6, 102]; MK801, 361 [−663, 2100]; and scopolamine, 44.7 [−25.6, 117]) with NAT-1 attachments in all mice (Figure 3f).

The trend of activity in the MK801- and scopolamine-treated animals (relative to the saline-treated mice) is robust for both experimental groups. Within-experiment analyses also showed that the MK801-treated animals had decreased global activity in the open field (relative to the saline-treated animals; Table S2), independent of NAT-1 attachments. The contrary was observed for the scopolamine-treated animals.

## 4. Discussion

The primary objective of this study was to evaluate the robustness of behavioural findings between animals with and without head stage/NAT-1 attachments in the open field. This device differs in many respects from other implantable miniaturised telemetric EEG recording systems for mice [36,37,38,39] and is considered an advancement over many tethered recording devices (see [8] for discussion). While many of these systems are widely used, a comparison between unoperated and surgically operated and animals is not commonplace but important to define the robustness of behaviours under scrutiny. For the open field, the behavioural repertoire in saline-treated animals was shown to be generally maintained across experimental groups with minimal implications of the surgery, head-stage moulding, and NAT-1 attachments. Animals treated with MK801 or scopolamine on the other hand responded to these modifications with an overall lowering of locomotor activity, with less wall hugging and more frequent visits to the centre of the arena. This suggests that despite considerable weight, NAT-1 per se does not induce behavioural changes, but these can be visualised in the presence of drugs. Such behavioural anomalies may include stereotypy, but most obviously were reflected in hypoactivity in this study. Despite wider confidence intervals in the NAT-1 cohorts treated with either MK801 or scopolamine treatment, the magnitudes of the differences between the drug groups and the controls were in the same direction and numerically very similar, supporting the robustness of the data and the resilience of the mice against neurosurgical intervention and weight-bearing head-attached devices. We note that these experiments were performed with a single dose of each drug, and behavioural anomalies may differ if higher or lower doses are administered. Nevertheless, the current results provide confidence that the utility of different doses would also lead to identical outcomes in non-operated and operated animals alike.

We therefore confirm that lightweight untethered head-stage/NAT-1 attachments do not impose serious limitations on locomotor activity—the primary outcome commonly assessed in the open field. However, there were variations pertaining to parameters of movement types and location-specific proxies, particularly in the thigmotaxic zone. Differences in location-specific proxies can potentially be explained by the animals’ somatosensory perception of the device on their head (‘object permanence’; [40,41]. The device may present a steric hindrance for the mobility of the animals as indicated by reductions in thigmotaxis measures in the wall zone of the arena. Other non-prespecified endpoints in this study showed variable NAT-1 attachment-dependent differences and included parameters of ethological values such as *freezing* or *heading error*, *the zone which was first entered*, *path efficiency to zone*, and *corrected integrated path length*. Intriguingly, drug-dependent changes in these proxies were not influenced by NAT-1 attachments. A more high-resolution analysis looking at shorter fragments of behavioural bouts [42] may return some differences, and their behavioural meaning requires determination.

Surgical intervention in Experiment 2 is arguably the biggest differential that may contribute to irreproducibility in behavioural outcomes between groups. It is imperative to consider possible side effects of the implanted electrodes or surgical process which may provoke changes in sleep pattern [24]; induce meningeal lymphangiogenesis and affect brain homeostasis [43]; or increase microglial activation and seizure susceptibility [21]. Weight bearing and drug treatment may further exacerbate such side effects (see [44] for discussion). What appears crucial is that a direct comparison between unoperated and surgically operated specimens with telemetric devices should be performed for each behavioural test before animals with EEG recording devices can be classed as ‘normal’ controls. There is still abundant room for further assessments to determine if responses remain robust in more complex behavioural paradigms or in, e.g., other mouse lines or genetically modified strains. An aspect that may be addressed in the future is the effect of head-stage/NAT-1 attachments on more fine-grained behavioural fractionations using unsupervised, data-driven approaches [42,45] which require considerable hardware and software upgrades. This may be relevant to studies exploring severe behavioural anomalies in different etiological responses as indications of anxiety [46,47,48] or epilepsy [49].

Overall, our data provide compelling evidence for by and large undisturbed behaviour in terms of spatial exploration, as all the changes induced by the drugs in the reported parameters were similarly observed with and without NAT-1 attachment. This study confirms the robustness of behavioural responses in the context of using wireless recording devices in the open field. Data robustness per se is important, as many laboratories provide EEG findings aligned with behavioural responses, such as ours [3,7,50]. Normative behaviour is critical if EEG recordings are interpreted as representative for a ‘normal’ animal.

## Figures and Tables

**Table 1:** Details pertaining to the study designs, experimental conditions, and husbandry in Experiments 1 and 2.

	Experiment 1	Experiment 2
**Study Outcome**	Pharmacological studies without EEG attachments	Pharmacological studies in animals with EEG attachments
**Persons involved (Blinder and experimenter)**	2	3
**Animals**		
**Strain**	C57BL/6J (Wildtype; total n = 29)	C57BL/6J (Wildtype; total n = 37; lost 10, see Section 2.3)
**Supplier/ Breeder**	Charles River Laboratories, Kent, UK	Charles River Laboratories, Kent, UK
**Transport**	By road	By road
**Specific pathogen free conditions**	Yes	Yes
**Age at arrival and acclimatisation to facility**	10 weeks; 2 weeks of acclimatisation	9 weeks; 2 weeks of acclimatisation
**Housing/ husbandry; test room conditions**		
**Cage type**	Conventional shoe box cages with wire top feeders; single housing	Conventional shoe box cages with wire top feeders (only 1 was moved to IVC cage); single housing
**Enrichment; bedding**	Cardboard rolls in all cages; corncob and aspen shavings (ratio 3:1)	No enrichment; corncob and aspen shavings (ratio 3:1)
**Temperature, humidity**	21 ± 2 °C; 50 ± 5%	21 ± 2 °C; 50 ± 5%
**Exposure of animal to handling**	Two/three times a day by facility staff	Two/three times a day by facility staff
**Noise/background noise**	Background noise (music) during light phase	Background noise (music) during light phase
**Dark/light cycle**	12:12 h; start of light phase at 07:00, no red light during dark phase	12:12 h; start of light phase at 07:00, no red light during dark phase
**Diet**	Normal chow pellets (SDS CRM) and room-temperature water ad libitum	Normal chow pellets (SDS CRM) and room-temperature water ad libitum
**Handling of cages**	Cleaning once every 2 weeks; water and food changed/topped up twice a week.	Cleaning once every 2 weeks; water and food changed/topped up twice a week.
**Experimental: animals**		
**Age of animal at start to experiment**	12–13 weeks (at the start of testing)	10–11 weeks (at surgery) and 12–13 weeks (at the start of testing)
**Transport of animals in facility**	Silent trolley type; cages were not blacked out or covered during transport	Silent trolley type; cages were not blacked out or covered during transport
**Time of testing, day of week**	Commencement at 10:00; testing was conducted from Monday to Thursday on the first week, and Monday to Wednesday on the second week.	Commencement at 10:00; testing was conducted from Monday to Thursday on the first week, and Monday to Wednesday on the second week.
**Total duration of test phase per day**	Approx. 5 h (4 animals tested am, 3 tested pm)	Approx. 5 h (3 animals tested am, 3 tested pm)
**Animal under diet restrictions during experimental phase**	No	No, except during NAT-1 application and drug administration: water bottles were removed, and cage lids were overturned to prevent NAT devices from accidentally wedging onto cage-lids and food-hoppers
* Naivety to experimental paradigm and drugs	Naïve to both experimental procedures and drugs	Home cage EEG recording and check, 24 h prior to open field test; naïve to drugs
**Experimental: surgical protocols**		
* Time period between surgeries and start of experiment	N.A.	2 weeks
* Total duration of surgery per day	N.A.	Approx. 7 h/day (commencement at 08.00; surgeries performed only on Mondays and Tuesdays
* Anaesthetic	N.A.	Gas anaesthesia (isoflurane) 1.0 mL saline (intraperitoneal) and 0.2 mL of 0.1 mg/kg buprenorphine (subcutaneous; Vetergesic^®^, Cera Animal Health Ltd., High Wycombe, UK).
* Analgesia	N.A.	Drinking water was supplemented with Rimadyl^®^ (Zoetis UK Ltd., Surrey, UK) for 2 days before and after the surgery
* Surgical procedure	N.A.	1 surgeon; 2 assistants
**Experimental: design and methods/data analysis**		
**Drug, and concentration**	0.9% saline; 0.65 mg/kg MK801; 0.85 mg/kg scopolamine. Intra-peritoneal administration of 10 mg/kg of treatment.	0.9% saline; 0.65 mg/kg MK801; 0.85 mg/kg scopolamine. Intra-peritoneal administration of 10 mg/kg of treatment.
* Experimenter	1 female	2 females and 1 male
* Frequency of handling	Handling during drug injections, immediately before and after test.	Handling during NAT-1 applications, drug injections, immediately before and after test.
**Randomisation method for allocation of animals to test groups**	random.org (https://www.random.org/sequences/, accessed 29 October 2018; Williams square design.	random.org (https://www.random.org/sequences/, accessed 19 February 2018); Williams square design.
**Blinding of experimenter to group allocation**	Yes.	Yes.
**Standardisation of animal handling across experimenters (protocol and training)**	Yes (1 in randomisation and blinding, 1 in drug administration and experiment)	Yes (2 involved in NAT-1 device application and drug administration and randomisation, 1 in experiment)
**Software used in recording and data extraction**	Automatic tracking software, ANY-Maze (v.5.1)	Automatic tracking software, ANY-Maze (v.5.1)
**Inclusion and exclusion criteria**	No inclusion criteria. Exclusion criteria based on analysis using Grubb’s method before re-evaluation of animal behaviour (video).	No inclusion criteria. Exclusion criteria based on analysis using Grubb’s method before re-evaluation of animal behaviour (video).
**Statistical analysis**	Bootstrapped one-way ANOVA and *t*-test for conventional analysis; repeated measures ANOVA for binned analysis; and estimation statistics to determine the magnitude of differences.	Bootstrapped one-way ANOVA and *t*-test for conventional analysis; repeated measures ANOVA for binned analysis; and estimation statistics to determine the magnitude of differences.
**Experimental: complexity of test arena**		
**Test arena**	Square; 50 × 50 × 50 cm; white Perspex	Square; 50 × 50 × 50 cm; white Perspex
**Average light intensity in test arena**	320 lux	320 lux
**Environment in test room (temperature, humidity and decibels relative to the carrier)**	20 ± 2 °C; 48 ± 5%; 30 ± 5 dBc; dedicted sound-attenuated room	20 ± 2 °C; 48 ± 5%; 30 ± 5 dBc; dedicted sound-attenuated room
**Number of test arenas used at any given time**	1	1
**Cleaning agents**	Non-fragrant and alcohol-free wet wipes	Non-fragrant and alcohol-free wet wipes

* Inconsistent experimental factors which differed between experiments.

**Table 2:** Summary of between-experiment comparisons for each drug treatment in pre-selected parameters. Data were obtained by bootstrapped independent *t*-tests (two-tailed), and mean difference was obtained using estimation analysis. All analyses were resampled 1000 times (seed starting at 123456) and obtained in R (v.4.3.0), and significance was considered where *p* < 0.05 (highlighted in red).

Parameters	Drug	Between-Experiment Comparisons			E2 minus E1
		E1	E2	*p-*Value	Mean Difference [95% CI]
		Mean ± SD	Mean ± SD		
**Distance moved (m)**	**Saline**	131.6 ± 20.2	139.9 ± 19.2	0.347	8.4 [−8.1; 24.6]
	**MK801**	70.0 ± 25.6	39.3 ± 23.5	0.028	−30.6 [−48.6; −6.0]
	**Scopolamine**	182.2 ± 20.0	155.2 ± 26.7	0.048	−26.9 [−52.3; −10.4]
**Average distance from** **centre-point (cm)**	**Saline**	0.218 ± 0.007	0.199 ± 0.010	0.001	−0.020 [−0.026; −0.012]
	**MK801**	0.155 ± 0.030	0.161 ± 0.050	0.782	0.006 [−0.041; 0.036]
	**Scopolamine**	0.237 ± 0.013	0.230 ± 0.012	0.275	−0.008 [−0.018; 0.003]
**Thigmotaxis (ratio)**	**Saline**	0.532 ± 0.052	0.463 ± 0.049	0.020	−0.068 [−0.11; −0.021]
	**MK801**	0.223 ± 0.157	0.285 ± 0.189	0.471	0.061 [−0.1; 0.207]
	**Scopolamine**	0.807 ± 0.147	0.777 ± 0.128	0.667	−0.030 [−0.131; 0.115]
**Thigmotaxis (s)**	**Saline**	1266.1 ± 96.3	1050.0 ± 105.7	0.002	−216 [−295; −123]
	**MK801**	545.1 ± 379.3	699.2 ± 445.6	0.435	154 [−199; 527]
	**Scopolamine**	1539.8 ± 184.5	1475.8 ± 163.8	0.437	−64 [−192; 107]
**Line-crossings**	**Saline**	1060.6 ± 145.3	1030.0 ± 132.49	0.442	−28.6 [−148; 86.7]
	**MK801**	866.9 ± 265.1	458.3 ± 238.5	0.014	−409 [−600; −158]
	**Scopolamine**	1374.2 ± 154.3	1012.0 ± 196.9	0.003	−362 [−541; −242]
**Meandering (degrees/s)**	**Saline**	912.5 ± 89.5	932.0 ± 107.4	0.668	19.5 [−63.6; 102]
	**MK801**	2716.4 ± 1008.4	3077 ± 1813.4	0.603	361 [−663.0; 2100]
	**Scopolamine**	705.6 ± 73.5	750.4 ± 89.2	0.325	44.7 [−25.6; 117]
**Rotation**	**Saline**	76.5 ± 10.5	98.8 ± 12.5	0.006	16.3 [5.6; 26]
	**MK801**	180.1 ± 142.7	158.2 ± 138.3	0.758	−21.9 [−156.0; 87.5]
	**Scopolamine**	99.8 ± 10.6	96.1 ± 18.2	0.623	−3.7 [−18.9; 7.16]

Thigmotaxis ratio was calculated by thigmotaxis distance divided by total distance moved; *SD*, standard deviation; CI, confidence intervals (upper, lower bounds); *E1*, Experiment 1 with no head-stage/NAT-1; and *E2*, Experiment 2 with head-stage/NAT-1.

## Data Availability

All data are presented in this paper and numerically will be made available on request.

## Supplementary Materials

The following supporting information can be downloaded at: https://www.mdpi.com/article/10.3390/biom14081008/s1, Figure S1: NAT-1 devise detailing dimensions of the microchip and positioning on a surgically operated animal. Table S1: List of obtainable parameters in ANY-Maze with their associated p-values from one-way analysis of variance and post-hoc analysis with Bonferroni’s corrections (between-drug comparisons in each experiment) and independent T-test analysis (between-experiment comparisons; corresponding heat-map found in Figure 1). Significant associations were p < 0.005 are highlighted in red and significant associations common to both experiments are highlighted in red and bolded. Statistical analyses were obtained in R (v.4.3.0). Table S2: Statistical summary of comparisons between treatment groups in Experiments 1 and 2 in pre-selected parameters. Conventional statistical comparisons against saline which differ between experimental groups are highlighted in red- significance is considered where *p* < 0.05. All statistical analysis for comparisons to saline in each experimental group were resampled 1000 (seed starting at 123456) in R (v.4.3.0).
