# Supplementary material for: Mouse Exploratory Behaviour in the Open Field with and without NAT-1 EEG Device: Effects of MK801 and Scopolamine
Source: Biomolecules. 2024 Aug 15;14(8):1008. doi: 10.3390/biom14081008 (PMC11352671; doi:10.3390/biom14081008)
Supplement: Supplementary file 1 [file biomolecules-14-01008-s001.zip › biomolecules-3131243-supplementary.pdf]

Supplement Figures

Figure S1: NAT-1 devise detailing dimensions of the microchip and positioning on a surgically operated animal.

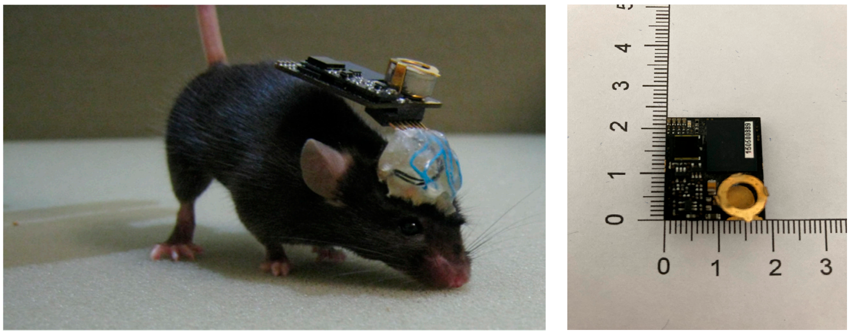

Supplement Tables

Table S1: List of obtainable parameters in ANY-Maze with their associated *p*-values from one-way analysis of variance and post-hoc analysis with Bonferroni's corrections (between-drug comparisons in each experiment) and independent T-test analysis (between-experiment comparisons; corresponding heat-map found in Figure 1). Significant associations were $p<0.005$ are highlighted in red and significant associations common to both experiments are highlighted in red and bolded. Statistical analyses were obtained in R (v.4.3.0).

Number and parameter name		Between-drug comparisons[a]				Between-experiment comparisons[b]		
		Experiment 1		Experiment 2				
		MK801 (ref. saline)	Scopolamine (ref. saline)	MK801 (ref. saline)	Scopolamine (ref. saline)	Saline	MK801	Scopolamine
**Apparatus measure**								
1	Duration	1.000	1.000	1.000	1.000	1.000	1.000	1.000
2	Distance	0.000	0.000	0.000	0.531	0.347	0.028	0.048
3	Mean speed	0.000	0.000	0.000	0.493	0.357	0.029	0.050
4	Max speed	0.000	1.000	0.000	1.000	0.001	0.130	0.004
5	Freezing episodes	0.064	0.136	0.042	1.000	0.079	1.000	1.000
6	Time freezing	0.120	0.263	0.481	1.000	0.077	0.553	0.430
7	Freezing latency	1.000	1.000	0.690	1.000	0.732	0.239	0.359
8	Mean freezing score	0.938	1.000	0.002	1.000	0.224	0.001	0.087
9	Time mobile	0.003	0.680	0.154	1.000	0.006	0.060	0.944
10	Time immobile	0.003	0.680	0.154	1.000	0.006	0.060	0.944
11	Mobile episodes	0.000	0.259	1.000	0.792	0.002	0.032	0.274
12	Immobile episodes	0.000	0.264	1.000	0.776	0.001	0.034	1.000
13	Mobility latency	0.747	1.000	1.000	0.142	1.000	1.000	0.305
14	Immobility latency	0.039	1.000	1.000	0.479	0.540	0.113	0.285
15	Rotations	0.027	1.000	0.296	1.000	0.006	0.758	0.623
16	Clockwise rotations	0.533	1.000	0.016	1.000	0.037	0.236	1.000
17	Anti-clockwise rotations	0.363	1.000	0.107	1.000	0.999	0.765	0.157
18	Absolute turn angle	0.003	1.000	0.276	1.000	0.064	0.029	0.141
19	Path efficiency	0.041	0.883	0.015	1.000	1.000	1.000	1.000
20	Line crossings	0.178	0.010	0.000	0.004	0.007	0.094	1.000
**16-grid measure**								
21	16-grid crossings	0.114	0.004	0.000	1.000	0.625	0.014	0.003
**Centre-point measures**								
22	Mean distance from	0.000	0.085	0.037	0.105	0.002	0.789	1.000
23	Max. distance from	0.000	0.085	0.037	0.105	0.001	1.000	0.275
24	Min. distance from	0.000	0.127	0.024	1.000	0.003	0.559	0.018
25	Time moving towards	0.149	0.000	0.001	0.000	0.618	0.265	0.655
26	Time moving away	0.001	0.037	1.000	0.000	0.023	0.149	0.704
27	Mean speed moving towards	0.897	1.000	0.000	0.429	0.075	0.024	1.000
28	Initial heading error	0.395	1.000	1.000	1.000	1.000	0.059	0.599
29	Average absolute heading error	0.000	0.030	0.071	0.325	1.000	1.000	1.000

**Thigmotaxis measures**								
**30**	Entries	0.174	0.010	0.000	0.005	0.007	0.093	1.000
**31**	Number exits	0.179	0.010	0.000	0.004	0.007	0.094	0.550
**32**	Time	0.000	0.055	0.042	0.011	0.002	0.435	0.442
**33**	Was 1st zone	1.000	1.000	0.698	1.000	1.000	0.151	1.000
**34**	Distance	**0.000**	**0.000**	**0.000**	**0.000**	0.448	0.279	0.067
**35**	Distance to first entry	0.166	1.000	0.019	0.787	0.141	0.611	0.218
**36**	Latency to first entry	0.232	1.000	0.012	1.000	0.013	0.641	0.063
**37**	Latency to first exit	0.334	1.000	0.016	1.000	0.565	0.618	0.026
**38**	Latency to last entry	0.211	0.478	0.567	1.000	0.656	0.443	0.182
**39**	Average speed	**0.001**	**0.000**	**0.000**	**0.007**	0.163	0.063	0.094
**40**	Max. speed	0.000	0.860	0.000	1.000	0.020	0.729	0.018
**41**	Max. visit	1.000	0.499	0.184	0.108	1.000	0.541	0.444
**42**	Min. visit	1.000	0.721	1.000	1.000	1.000	1.000	1.000
**43**	Mean visit	0.922	0.003	1.000	0.102	0.009	0.124	1.000
**44**	Time mobile	**0.000**	**0.002**	**0.000**	**0.001**	0.218	0.756	0.318
**45**	Time immobile	0.008	1.000	0.856	0.400	0.004	0.169	0.984
**46**	Immobile episodes	0.000	0.839	0.069	0.874	0.015	0.081	1.000
**47**	Initial distance	0.462	1.000	1.000	0.260	0.033	0.099	0.002
**48**	Mean distance from	0.000	1.000	0.031	1.000	0.001	1.000	1.000
**49**	Max. distance from	1.000	0.055	1.000	0.249	1.000	1.000	0.147
**50**	Min. distance from	1.000	1.000	0.698	1.000	1.000	0.151	1.000
**51**	Cumulative distance	0.000	0.339	0.042	0.497	0.001	0.833	0.326
**52**	Mean distance to border	0.023	0.319	1.000	0.432	1.000	1.000	1.000
**53**	Max. distance to border	0.029	1.000	0.349	1.000	1.000	1.000	1.000
**54**	Min. distance to border	1.000	1.000	1.000	1.000	1.000	1.000	1.000
**55**	Time getting closer to zone	0.232	1.000	0.230	1.000	0.014	0.414	0.043
**56**	Time getting further from zone	0.253	1.000	0.248	1.000	0.057	0.423	0.101
**57**	Initial heading error	0.277	0.953	1.000	1`.000	0.868	0.144	0.416
**58**	Signed initial heading error	1.000	1.000	1.000	1.000	0.563	0.711	0.514
**59**	Average absolute heading error	**0.001**	**0.001**	**0.000**	**0.000**	1.000	1.000	1.000
**60**	Time moving towards	0.021	0.000	0.893	0.000	0.850	0.350	0.636
**61**	Time moving away from	0.000	0.844	0.093	0.016	0.011	0.253	0.497
**62**	In zone oriented to centre	0.005	0.005	0.354	0.467	0.867	0.480	0.158
**63**	Absolute turn angle	0.000	0.009	0.000	0.073	0.226	0.208	0.040
**64**	Freezing episodes	0.049	0.131	0.562	1.000	0.061	1.000	0.399
**65**	Time freezing	0.131	0.296	1.000	0.515	0.066	0.612	0.532
**66**	Path efficiency to entry	0.523	0.686	0.241	1.000	0.991	0.746	0.480
**67**	Corrected integrated path length	0.000	0.336	0.040	0.464	0.001	0.858	0.348
**68**	Number of line crossings	0.177	0.010	0.000	0.005	0.007	0.093	1.000

[a]Analysis of variance with post-hoc Bonferroni's corrections. [b]Independent T-test analysis, associated heat-map representations in Figure 2. *Experiment 1*, with no head-stage/NAT-1; *Experiment 2*, with head-stage/NAT-1; ref., referenced to; Max., maximum; Min., minimum.

Table S2 Statistical summary of comparisons between treatment groups in Experiments 1 and 2 in pre-selected parameters. Conventional statistical comparisons against saline which differ between experimental groups are highlighted in red- significance is considered where p<0.05. All statistical analysis for comparisons to saline in each experimental group were resampled 1000 (seed starting at 123456) in R (v.4.3.0).

Parameters	Within-experiment comparisons[a]						MK801 minus saline		Scopolamine minus saline	
	Saline	MK801	Scopolamine	F[b]	Sig.	ηp^2				
	mean±SD	mean±SD	mean±SD				Mean difference [95% CI]	p^a	Mean difference [95% CI]	p^a
**Distance moved (m)**										
**E1**	131.6±20.2	70.0±25.6	182.2±20.0	61.9	<0.001	0.826	-61.6 [-81.5; -42.2]	<0.001	50.6 [34.1; 68]	<0.001
**E2**	139.9±19.2	39.3±23.5	155.2±26.7	65.3	<0.001	0.845	-101 [-120; -79.7]	<0.001	15.3 [-8.76; 32.5]	0.531
**Average distance from centre-point (m)**										
**E1**	0.218±0.007	0.155±0.03	0.237±0.013	48.9	<0.001	0.790	-0.065 [-0.080; -0.044]	<0.001	0.020 [0.008; 0.026]	0.085
**E2**	0.199±0.01	0.161±0.050	0.230±0.012	12.2	<0.001	0.505	-0.039 [-0.082; -0.014]	0.037	0.032 [0.019; 0.042]	0.105
**Thigmotaxis (ratio)**										
**E1**	0.532±0.052	0.223±0.157	0.807±0.147	50.5	<0.001	0.795	-0.308 [-0.403; -0.201]	<0.001	0.278 [0.118; 0.339]	<0.001
**E2**	0.463±0.049	0.285±0.189	0.777±0.128	30.7	<0.001	0.710	-0.179 [-0.304; -0.062]	0.038	0.316 [0.219; 0.394]	<0.001
**Thigmotaxis (s)**										
**E1**	1266.1±96.3	545.1±379.3	1539.8±184.5	41.7	<0.001	0.762	-721 [-927; -473]	<0.001	274 [95.9; 363]	0.055
**E2**	1050.0±105.7	699.2±445.6	1475.8±163.8	17.3	<0.001	0.590	-351 [-623; -75.8]	0.042	426 [280; 538]	0.011
**Line-crossings**										
**E1**	1060.6±145.3	866.9±265.1	1374.2±154.3	16.9	<0.001	0.565	-194 [-403; -19.2]	0.114	314 [187; 440]	0.004
**E2**	1030.0±132.49	458.3±238.5	1012.0±196.9	25.4	<0.001	0.679	-574 [-724; -371]	<0.001	-20 [-196; 97.6]	>0.999
**Meandering (degrees/m)**										
**E1**	912.5±89.5	2716.4±1008.4	705.6±73.5	35.9	<0.001	0.734	1800 [1260; 2480]	<0.001	-207 [-277; -142]	>0.999
**E2**	932.0±107.4	3077±1813.4	750.4±89.2	13.7	<0.001	0.533	2150 [1330; 3860]	0.001	-182 [-264; -98.3]	>0.999
**Rotation**										
**E1**	76.5±10.5	180.1±142.7	99.8±10.6	4.4	0.023	0.251	104 [27.4; 209]	0.027	23.3 [14.7; 32.1]	>0.999
**E2**	98.8±12.5	158.2±138.3	96.1±18.2	1.9	0.176	0.135	65.4 [-0.778; 175]	0.293	3.33 [-11.3; 15.6]	>0.999

[a]Bootstrapped analysis of variance with Bonferroni's corrections. [b]Experiment 1, F(2,26); Experiment 2, F(2,24). *SD*, standard deviation; *F*, *f*-values; sig., *p*-values with significance levels set at *p*<0.05; *ηp2*, partial eta-squared; CI, confidence intervals (lower; upper bounds); E*1*, Experiment 1 with no head-stage/NAT-1; and *E2*, Experiment 2 with head-stage/NAT-1.
